# Building recovery capital: co-creating an assets-based community engagement toolkit for people accessing drug and alcohol treatment services

**DOI:** 10.3389/fpubh.2026.1965897

**Published:** 2026-09-29

**Authors:** Amy Beardmore, Devon De Silva, Leila Powell

**Affiliations:** 1 Center for Public Health and Wellbeing, School of Health and Social Wellbeing, University of the West of England, Bristol, United Kingdom; 2 Via, Innovation and Research Unit, London, United Kingdom

**Keywords:** assertive linkage, community assets, community engagement, drugs and alcohol, recovery capital

## Abstract

**Background:**

Community assets such as social groups, volunteering opportunities and wellbeing activities can strengthen recovery capital and support long-term recovery; however, people accessing drug and alcohol treatment services often face barriers to engagement. This study aimed to co-create an Assets-Based Community Engagement (ABCE) toolkit to strengthen connections between treatment services and community assets.

**Methods:**

A rapid evidence review informed a 12-week co-creation project involving workshops with service users, peer mentors and staff. Participants explored barriers, enablers and motivators to community asset engagement and identified priorities for toolkit development. Data were analyzed using a priori coding.

**Results:**

Key barriers included cost, transport difficulties, digital exclusion, low confidence and limited awareness of opportunities. Enablers were peer support, trusted relationships, practical guidance and accessibility, while motivators centered on wellbeing, social connection and personal development. The co-creation process produced four toolkit components: a six-stage engagement model, a Community Connector role description, a guided conversation tool and recommendations for enhancing a digital community assets platform.

**Discussion:**

Findings highlight the importance of addressing practical, social, and informational barriers to community engagement and demonstrate how rapid co-creation can generate practical resources for treatment services.

**Conclusion:**

The ABCE toolkit offers a scalable, recovery-oriented framework for strengthening recovery capital through increased engagement with community assets and embedding asset-based practice within treatment and recovery services.

## 1 Introduction

For individuals experiencing drug and alcohol dependency, accessing treatment is a critical first step toward recovery. Yet recovery extends beyond cessation, and achieving sustained recovery frequently requires significant social and lifestyle changes, including the development of new social networks and interpersonal relationships that support the reconstruction of an identity independent of substance use. Such identity transformation is increasingly recognized as a central component of long-term recovery and recovery maintenance (1, 2).

However, efforts to reintegrate into community life and establish new social connections may also expose individuals to stigma associated with their previous substance use. This can result in experiences of social exclusion and marginalization, with detrimental consequences for emotional wellbeing and health outcomes (3). Given that stigma can negatively affect perceptions of treatment, impede recovery processes, and increase the risk of relapse (4, 5), it is crucial that opportunities for community participation are experienced as inclusive, meaningful and sustainable over the long term.

It is known that establishing a connection to community activities and engaging with local organizations can support personal development and nurture positive behaviors, which can in turn greatly assist in achieving long-term successful health outcomes (6). Community assets that are supportive of those in recovery can play a significant role in helping people navigate this process by meeting their immediate lifestyle needs (for example, debt or housing advice) and by giving them access to a wide variety of social and community activities and support (such as choirs, physical activity or art groups) when they feel ready and able to do so.

However, precisely *how* people discover and then effectively access community assets in support of their recovery remains unclear, as do the mechanisms by which meaningful engagement is successfully sustained over time. In this paper we present a co-created toolkit that aims to address these issues, the purpose of which is to help treatment providers effectively link people using their services with community assets in a way that is both appropriate to their current stage of recovery and commensurate with the individual's needs and wants.

Drawing on models of social prescribing and the Transtheoretical Model (TTM) [otherwise known as the “Stages of Change” – (7, 8)], the toolkit suggests several ways of effectively linking people in recovery to community assets. Two notable elements include our new “Stages of Engagement” framework and a community connector person specification, tailored to recovery services. The toolkit will be of use to anyone working in drug and alcohol treatment services but also has applications across a range of settings where strengths-based approaches are operationalised.

Understanding the factors that support sustained recovery from substance use is central to the development of effective interventions. Contemporary recovery approaches increasingly emphasize holistic and person-centered support, recognizing that recovery extends beyond abstinence to encompass broader aspects of health, wellbeing and social functioning. Maslow's Hierarchy of Needs (9) provides a useful framework for understanding these wider recovery processes. The model proposes that human motivation is shaped by a progression of needs, beginning with physiological and safety requirements and extending to belonging, esteem and self-actualisation.

The hierarchy highlights the importance of social relationships and a sense of belonging in promoting wellbeing and personal development. Meeting psychosocial needs is an important foundation for recovery, but for individuals to thrive they need to be able to access the additional benefits that come with having self-esteem and achieving personal growth. Consequently, the framework has been applied within addiction and recovery settings to support person-centered care planning that responds to individuals' changing needs over time (10). This broader understanding of recovery provides a rationale for exploring the role of community assets in supporting social connectedness, personal growth, and long-term recovery outcomes.

Community assets can be defined as the services, spaces and opportunities that exist within a local area that contribute to people's health and wellbeing (11). These assets provide different forms of support depending on the needs and wants of an individual; some offer practical help or specialist services, whilst others create opportunities for social interaction, skill-building and personal development. Together they form a network of support that people can draw on in their everyday lives, and accessing these assets can help individuals feel connected, supported and more confident, whilst also providing advice or assistance when needed (12).

The value of community assets in supporting recovery therefore extends beyond the immediate function of these assets or services by creating opportunities for social interaction, participation and civic engagement. If accessed successfully and at the right time in an individual's recovery journey, they can have a profound impact on personal and social development, often leading to positive identity shifts and changes in behavior at an individual level, whilst also having a positive impact on overall community connectedness (6).

Understanding how community assets contribute to recovery requires consideration of the social resources they help to generate and sustain. The concepts of social capital and recovery capital provide a useful framework for explaining how connections, relationships and access to resources influence health, wellbeing and long-term recovery. At the heart of social capital is the premise that the stronger our connections are to other people and the more sustainable those connections are over time, the easier it is to achieve our goals because we are more likely to have resources and support on which to draw in times of need (13).

Situating social capital within a drug and alcohol context, Granfield and Cloud (14) introduced the idea of “recovery capital,” a concept that draws on a variety of approaches, including “natural recovery, solution-focused therapy, strengths-based case management, recovery management, resilience and protective factors, and the ideas of hardiness, wellness, and global health” [(15), p.1]. The idea is straightforward: social ties, access to resources and connections to other people are especially important for people in recovery as they can help create new networks and relationships and boost the chances of staying well over time (16, 44).

The literature takes a primarily socioecological approach to recovery capital, focusing on three key elements: the individual/personal, the social, and the community (15). In Collinson and Best's (6) “ice-cream model” interpretation, recovery capital is built through increasing levels of community engagement, starting with personal recovery capital (skills, resilience, self-esteem, aspirations), followed by social recovery capital (supportive relationships, friends, family, recovery networks) and lastly community recovery capital (groups, activities, opportunities and access to community assets). Consequently, community assets can be understood as resources in their own right, but also as mechanisms through which recovery capital is generated. The additional insight that the authors offer with this model, however, is that there are also benefits for the wider community in terms of improved wellbeing – these are the “sprinkles” on top of the ice cream that result in greater community cohesion, stronger local networks and reduced stigma.

Assets Based Community Development (ABCD) has long recognized that these local resources, interpersonal relationships and tangible assets are crucial tools in building communities that can thrive (17). Rather than identifying what a community lacks, in ABCD a “glass half full” approach is taken, acknowledging that existing strengths are the building blocks for creating empowered and participatory communities (18). Adapting this approach to recovery, Collinson and Best (6) argue that there should be an additional focus on *how* people engage with these community assets – it is not enough, they say, to simply signpost people to community activities or hope they attend following a professional referral. They suggest instead that an Assets Based Community Engagement (ABCE) framework is utilized whereby assets, personal interests and barriers to engagement can be explored with the individual through discussion with an intermediary with lived experience, such as a community connector or peer mentor. The purpose of these conversations is to firstly identify assets, and secondly “to act as a human bridge to encourage people in recovery to engage with those assets and groups” [(19), p.125] by sharing their own knowledge and lived experience and actively encouraging engagement. This process is known as assertive linkage (20).

Organizations have previously undertaken asset-mapping exercises (21) and developed toolkits to support meaningful engagement with communities (22). Similar asset-based approaches have also been used in other health and social care settings, including community mental health (23) and several toolkits exist within the context of broader community development initiatives (24–26). However, whilst these approaches offer practical guidance for identifying community assets and strengthening community connections, to the best of our knowledge there is currently no practical, recovery capital-informed toolkit designed specifically to support drug and alcohol treatment providers to assertively link service users with community activities in pursuit of meaningful and sustained recovery. This paper therefore presents a co-created toolkit developed for that purpose.

## 2 Materials and methods

### 2.1 Context and purpose

In November 2025, Via - a registered drug and alcohol charity that delivers services commissioned by the local authority – began working with a research team from the University of the West of England (UWE Bristol) on an accelerated knowledge transfer (AKT) partnership funded by Innovate UK. The AKT aimed to gain a better understanding of the barriers and facilitators to engagement with community assets for people living with dependency, with the objective of scoping a tool to aid linkage to assets in support of recovery.

Prior to the formation of the partnership, Via had developed a searchable online digital community assets platform (later named ‘*Via Connect'*) that was ready to be made available to service users. Whilst asset-mapping work had already been done during the creation of this resource, before the platform could go live Via wanted to understand *how* service users could be effectively and meaningfully linked to those assets, regardless of where they were in their recovery journey.

Drawing on the expertise of the UWE Bristol academics and the operational knowledge of the Via team, a project plan was finalized based on the initial project proposal and a co-creation methodology was agreed.

Through a process of co-creation with Via's peer mentors, staff and service users, the AKT sought to answer two key research questions:

1. How do we help people recognize what they need or want depending on where they are in their recovery journey?
2. How can we empower people to access and engage with assets in a meaningful and sustainable way?

The purpose of this paper is therefore 2-fold; first we discuss the *process* of co-creating the toolkit through a series of meetings and workshops with staff, peer mentors and service users; second, we present the toolkit itself.

### 2.2 Study design

Our study used an exploratory co-creation design to explore stakeholder perspectives and collaboratively develop insights for the purpose of the development of a practical toolkit.

Whilst co-creation shares several similarities with co-production approaches, we chose the former study design as our focus was not on implementation but rather on the toolkit design and associated the decision-making processes (27).

Although we built co-creation into every stage of the study as far as was possible, we were also aware that the rapid nature of the research meant that there were potential limitations to the depth of that involvement and that without careful planning our approach could be conceived as tokenistic. We were mindful therefore to ensure that the activities were engaging and felt meaningful to participants, and that time was built in for reflection at the beginning and end of each workshop (28).

Similarly, as with all co-produced or co-created work, we were careful to avoid participants becoming disillusioned or frustrated by the limitations of what was possible in the timeframe (29), however in many ways the rapid nature of the research helped with this as participants were able to see the results of their input within a matter of weeks.

### 2.3 Recruitment

An in-service purposive sampling approach was used to recruit participants to the meetings and workshops, whereby Via's peer mentors and key workers informed individuals about the study if they felt they would be suitable to participate according to their stage of recovery [it is important to note here that recovery journeys can be very complex and deeply personal, and that recovery doesn't always mean achieving total abstinence. For many, it is a multifaceted process which involves regaining control over problematic substance use, increased participation in community life and sustaining lifestyle changes over time (30). We therefore accepted anyone to the study who was engaging with Via's services, whether they were new to treatment or had been attending for some time]. This approach not only ensured that participants felt able to participate but also allowed workers to select experienced and information rich cases (31). Participant information and consent sheets were provided, and informed consent was obtained before participation in the meetings and workshops.

As is standard practice in drug and alcohol treatment services, individuals were offered an incentive to participate, in this case through Via's Capital Card benefit scheme which rewards engagement with redeemable points. These points can be spent in the Capital Card shop, for items like toiletries or DVDs, or accrued and used with external spend partners or for learning courses (32).

### 2.4 Project delivery

The project took place over a period of 12 weeks and ran from November 2025 – February 2026, including a one-week break for the Christmas vacation. A Research Associate (LP) had already been recruited prior to the start of the project and ethical approval granted over the summer, both of which aided our ability to start immediately; however, the short time frame remained an obvious challenge.

The project timeline is illustrated in Figure 1 and the key data collection points are described below.

**Figure 1:**
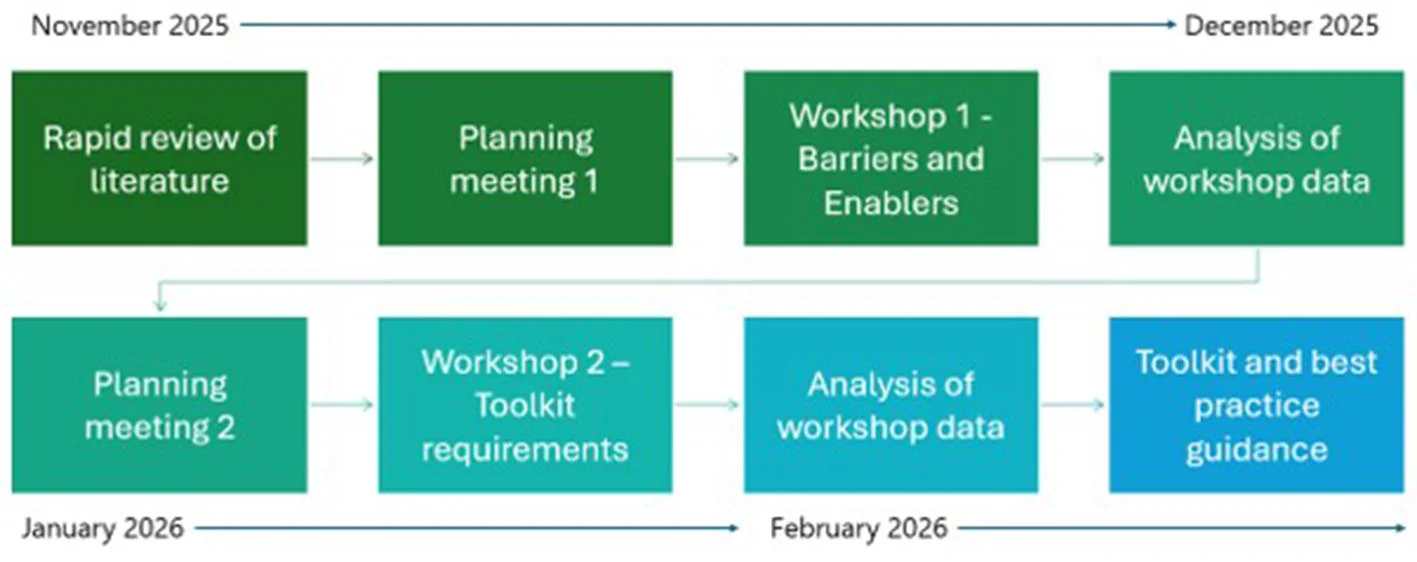
Methodological process.

#### 2.4.1 Literature review – November 2025

A desk-based review of the existing literature was conducted, revealing insights into assertive linkage and assets-based community engagement, both of which became integral to the theoretical basis of the project. A lay summary of this literature review was given to participants at the first workshop.

#### 2.4.2 Planning meeting 1 – November 2025

An initial project planning meeting was held in November 2025 with Via staff (*n* = 4), peer mentors (*n* = 3) and the UWE Bristol team (*n* = 2). Together, the group designed the content and established the aims of the first workshop. Guidance and training were also provided to the peer mentors during this session on co-facilitation.

#### 2.4.3 Workshop 1 – December 2025

The first workshop was held in early December 2025 and was attended by Via staff (*n* = 1), peer mentors (*n* = 3), service users (*n* = 6) and the UWE Bristol team (*n* = 2).

This workshop centered around an asset mapping exercise that sought to identify the barriers, enablers and motivators to engagement with community assets. Participants were asked to map out any assets that they were currently using, as well as those that they would like to engage with but haven't. This was followed by a facilitated discussion in which the answers were explored in more detail.

#### 2.4.4 Planning meeting 2 – January 2026

A second planning meeting was held in early January 2026 and was attended by Via staff (*n* = 1), peer mentors (*n* = 3) and UWE staff (*n* = 2). At this meeting participants were asked to make suggestions to name Via's digital community assets platform, based on key themes and words that had come up during the first workshop. These included: “Community Connection,” “Via Connect” and “Community Spirit,” and peer mentors were asked to collect votes from service users with regards to their preference before the second workshop.

Using the initial insights from the first workshop, the group planned workshop two activities, with a focus on toolkit design.

#### 2.4.5 Workshop 2 – January 2026

The second workshop was held in mid-January 2026 and was attended by Via staff (*n* = 4), peer mentors (*n* = 3), and UWE staff (*n* = 2).

Forty-two votes had been collected by peer mentors regarding the name of the digital platform, and with a total of 28 votes it was decided that the platform would be named “Via Connect.”

A summary of the findings from the first workshop was presented and a reflective discussion was held. This information was then used to explore what could go into the toolkit that would encourage engagement with community assets and Via Connect.

To help facilitate these discussions, fictional personas (Table 1) were used to identify points in a person's timeline where community assets could be discussed and what techniques or approaches could help with this. Again, this was followed by a facilitated discussion where these ideas were explored in more detail.

**Table 1:** Fictionalised service user personas.

Dave
*Gender*: Male
*Age*: 18
*Ethnicity*: White British
*Sexuality*: Not reported
*Family*: Boyfriend to a woman named Sarah
*Employment status:* Not reported
*Skills:* None listed
*Substance use:* Alcohol & heroin
*Mental health:* Not reported
*Goals:* None provided
*Motivations:* Housing
*Frustrations/personal difficulties*: Sleeping rough, frustrated that previous friends have direction and stability in their lives, struggled with anger management, lack housing opportunities, reliant on the services but resents their authority and own ability to be self-directed, struggled to get on with people his age
*Quotes: “I've never managed to get on with people my age...so yeah I was always out with boys that were like 16 and 17.”*
*Known interventions:* Opioid substitution therapy, outreach
Sam
*Gender:* Woman
*Age:* 50s
*Ethnicity:* South Asian
*Sexuality:* Straight
*Family:* Mother, Wife, Sister
*Employment status:* Former nurse
*Skills:* Artistic, kind when sober
*Substance use:* Alcohol
*Mental health:* Trauma
*Goals:* None provided
*Motivations:* Hindu
*Frustrations/personal difficulties:* Childhood abuse, becomes abusive to family when drunk, not willing to engage in support, criminal justice system (arrested/courts)
*Quotes:* None provided
*Known interventions:* None

### 2.5 Data collection

Data were collected during the in-person meetings and workshops using a range of different methods. This included the outputs from the asset-mapping and persona-mapping sheets, discussion notes completed by participants during the workshops, and notes and observations captured by the research team. Physical data (such as flip chart paper and sticky notes) were photographed and immediately uploaded to UWE's secure OneDrive before being deleted from the device. Alongside the notes, these were later analyzed to generate findings.

### 2.6 Data analysis

Given the rapid nature of the research, data were analyzed by LP using an a priori approach guided by predetermined themes that were explored through the workshops (barriers to accessing community assets, enablers to accessing community assets, motivators and tool development) with sub themes based on information gathered through the literature review (33). Workshop materials were reviewed systematically and annotated, with data segments coded into the categories. Emerging insights that did not fit the initial coding framework were developed into an additional category, allowing the analysis to capture unanticipated insights. The themes and data were then cross-referenced and checked by AB and DDS to ensure they were representative of the facilitated workshop discussions.

### 2.7 Ethical considerations

Ethical approval was granted by UWE Bristol's ethics committee on 25th September 2025 (application ID 14712833).

Because the research involved potentially vulnerable participants, information was provided in plain English, clearly explaining the study, participation requirements, confidentiality, anonymity and data processing. Participants could also discuss the research with their key worker or Via staff before deciding to take part.

Workshops were held at Via's local branch which provided a familiar and comfortable setting for participants. Activities were designed to allow participants to share their views through case studies rather than personal experiences if preferred, and icebreakers, check-ins and check-outs were used to foster a supportive environment that encouraged self-reflection.

## 3 Results

### 3.1 Workshop one – barriers, enablers and motivators

During our discussions in the first workshop, participants frequently reported engaging in free activities that were run by Via, including Zumba, art groups and food hygiene training. However, engagement with community assets beyond the service was limited, with cost, transport, lack of confidence and stigma frequently reported as significant barriers. That said, some participants reported enjoying playing guitar, swimming, skateboarding and attending local faith groups in their free time. They also expressed strong desires to engage in a broader range of activities in the future, including joining a gym and exercising “to feel good,” developing skills for employment, accessing financial advice and training.

The barriers, enablers and motivators identified by our participants in workshop one, are summarized in Tables 2–4 below, supported by direct quotes and general comments from participants.

**Table 2:** Reported barriers to engagement with community assets.

Barrier	Definition	Examples given	Illustrative quotes and examples
Structural barriers	Practical, environmental, or organizational obstacles that prevent service users from accessing community assets	Transport issues, operating hours, geographical distance, digital exclusion, capacity limits	“Everything I'd want to go to is two buses away, and I can't afford the fares” “Running club is £2 a week; I can afford the gym but not everyone can” “I don't have a bank account”
Service navigation barriers	Difficulties understanding or navigating community assets, pathways or referral processes	Confusion about steps, unclear signposting, inconsistent information	“I never know who to ask or how to get started with stuff outside Via” “Food bank – you need to know how to access it”
Stigma or social exclusion	Experiences of internalized stigma, discrimination, or marginalization that affects willingness to engage with community resources	Shame, fear of judgement, feeling unwelcome	“People will think badly of me if they know [about my substance use]” “There can be shame in accessing financial advice”
Limited awareness of community assets	Lack of knowledge about what community assets exist or what they offer	Not knowing where assets are, what they do, or who can access them	“There might be groups, but I've never been told about them”
Barriers to accessing digital or online resources	Challenges using digital resources	Lack of digital literacy, usability issues, accessibility issues, device limitations	“I tried, but there were too many steps and I got lost”
Lack of confidence or readiness	Emotional or psychological factors limiting engagement with community activities	Anxiety, self-doubt, readiness concerns, low motivation	“I wasn't ready to meet new people yet” “Meeting others for the first time can feel daunting”
Cultural or identity misalignment	Community assets or engagement processes that do not feel culturally safe or inclusive	Mismatch with cultural values, lack representation, identity-based exclusion	“I felt like the group didn't understand my background”

**Table 3:** Reported enablers for engagement with community assets.

Enabler	Definition	Examples given	Illustrative quotes and examples
Clear and supportive pathways	Straightforward, well-communicated routes to accessing community assets	Clear steps, streamlined referrals, structured engagement processes	Participants noted that encouragement from key workers and funding for some courses – such as food hygiene – were also helpful
Staff support and guidance	Practical and emotional support from staff to connect users with assets	Warm handovers, staff-led navigation, encouragement from workers	“We need humans [to help], not AI”
Peer support	Any non-specific help from peers with lived experience that facilitates engagement	Peer guidance, emotional support, shared experience	“Talking to someone who's been through it made me want to give it a go” “Peers have recommended Zumba and I'm going to go”
Community connectedness	Sense of belonging, social inclusion, or community integration that supports engagement	Feeling welcomed, accepted, valued	“Once I felt part of it, I kept going back”
Person-centered/asset-based approaches	Strengths-based or recovery-focused approaches that support engagement	Empowerment, focusing on strengths, values-driven support	“They helped me think about what I'm good at and what kind of groups suited me”

**Table 4:** Reported motivators for engagement with community assets.

Motivator	Definition	Examples given	Illustrative quotes and examples
Desire for recovery and wellbeing	Internal motivation to improve health, stability and personal wellbeing	Growth, change, desire for stability	“I wanted to feel healthier and get my life back”
Social motivation	Desire to connect, reduce isolation, or build new relationships	Wanting friends, belonging, or support networks	“I'm tired of feeling lonely all day” “I want to fill the day with more activities, learn about different topics and meet new people”
Skill-building and personal development	Motivation to learn, volunteer, work, or build competence	Job skills, creative hobbies, vocational goals	“I joined the group because I wanted to learn something new”
Perceived benefit	Belief that engaging with a specific asset will help recovery or quality of life	Expected outcomes	“I heard it really helps people in my situation”

### 3.2 Workshop two – identifying key elements of the toolkit

Workshop 2 generated detailed recommendations for the Community Connector role, associated training and the proposed service user pathway.

#### 3.2.1 Community connector role

Participants expressed the view that Community Connectors should be approachable, empathetic and emotionally intelligent individuals who could provide gradual, tailored support throughout recovery. The role was envisioned as being both relational and practical, requiring the ability to build confidence, create safe spaces, facilitate informal conversations and use lived experience to encourage engagement. Participants emphasized the importance of introducing Community Connectors early in the recovery journey and maintaining their involvement over time, with responsibilities including accompanying service users to community activities, developing links with local assets, facilitating drop-ins and supporting the ongoing development of the digital platform.

#### 3.2.2 Training requirements

Participants also identified key training requirements for Community Connectors. These included communication and de-escalation skills, understanding how to pace support according to an individual's stage of recovery and the ability to use strengths-based approaches to explore a person's interests, priorities, fears and motivations. Scenario-based learning, case studies and role-play exercises were suggested to support the development of rapport-building skills and confidence in managing real-world situations. Training on the use of the digital platform was also considered essential.

#### 3.2.3 Recovery pathways

Through a persona-mapping exercise, participants co-produced a staged recovery pathway. They emphasized that support should begin with understanding the individual's immediate needs and priorities, with community connections introduced gradually to avoid overwhelming new service users. Early engagement was seen as the responsibility of key workers, with Community Connectors becoming increasingly involved as confidence and recovery progressed. Participants highlighted the importance of regular reviews, access to practical and emotional support and opportunities for broader community participation, volunteering and peer support. Ultimately, they envisaged a pathway in which individuals move from supported engagement with community assets toward independent use of the digital platform, with some potentially progressing into Community Connector roles themselves.

### 3.3 The toolkit

Once data collection was complete, the UWE Bristol team analyzed the outputs and findings from the workshops and – in partnership with Via's Innovation and Research Unit Manager (DDS) - a practical toolkit was developed. The four elements of the toolkit are presented below.

#### 3.3.1 The stages of engagement framework

Originally developed to explain behavior change processes, Prochaska and DiClemente's Transtheoretical model – or the Stages of Change - (7) is widely used in health promotion and addiction services to support individuals seeking to modify behaviors that adversely affect their wellbeing. Although the model has been applied across a range of behavioral contexts, it is most associated with smoking cessation and recovery from substance use disorders (8).

Findings from consultations with service users and peer mentors suggested that engagement with community assets may follow a process analogous to the stages of change framework. Participants highlighted that an individual's willingness and capacity to access community-based resources often varies according to their stage of recovery. In response, the framework was adapted to conceptualize readiness for community engagement rather than readiness for recovery from substance dependence.

The adapted model (Figure 2) proposes that individuals move through different levels of preparedness to engage with community assets and that support strategies should therefore be tailored to their current stage of engagement. By recognizing an individual's level of readiness, practitioners and Community Connectors can provide appropriate support, address barriers to participation and build on existing strengths to promote autonomy, social connection and sustained involvement within the community. Importantly, the objective extends beyond initial attendance to fostering meaningful and enduring participation that contributes to wellbeing and a sense of belonging. A detailed explanation of each step of the framework and guidance for practitioners can be found in Table 5.

**Figure 2:**
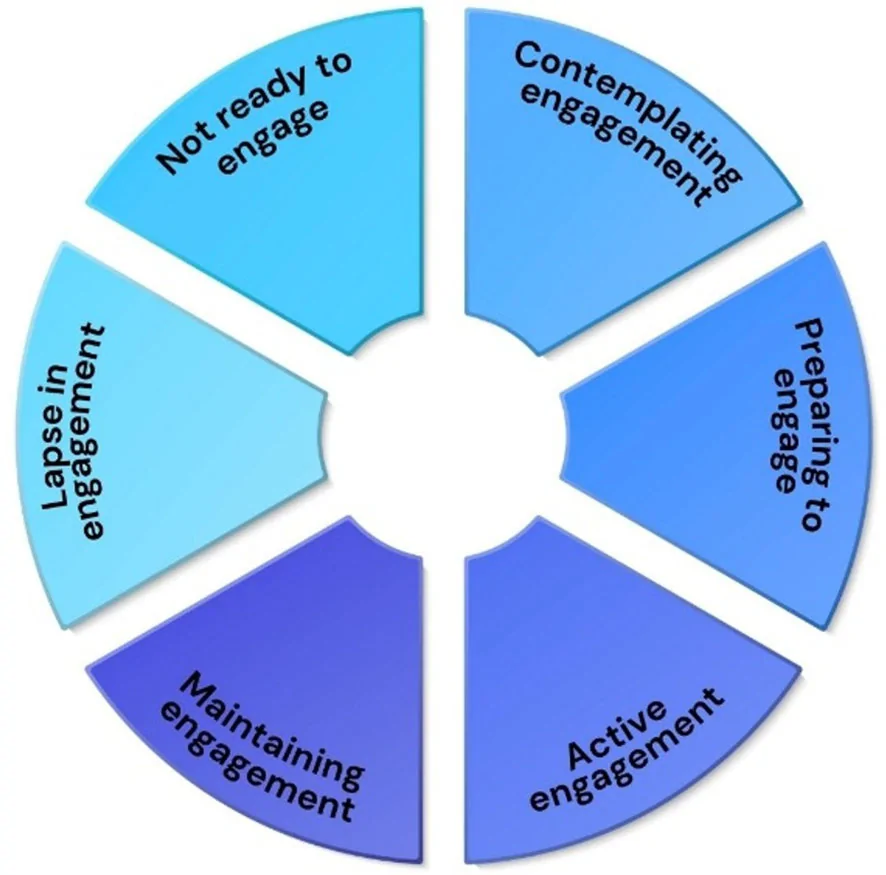
The stages of engagement framework.

**Table 5:** Steps in the stages of engagement framework.

Stage	Presentation	Goal	Approach	Tools
**Not ready to engage** (Not ready or interested)	• May not see assets as relevant. • May have no knowledge of assets. • May have negative past experiences. • May have low confidence, anxiety, limited awareness.	Increase openness and curiosity, raise awareness.	• Validate their concerns, avoid pushing. • Gently explore values (connection, health, purpose) rather than activities. • Assets such as debt or housing advice may be more appropriate than community activities at this stage.	• Marketing such as infographics, leaflets. • Light touch signposting and advice. • Guided conversation tool to assess interests and needs
**Contemplating engagement** (Maybe, but unsure)	• Recognizes potential benefits. • Concerns about barriers (costs, transport, social anxiety, energy).	Strengthen motivation, confidence and awareness	• Explore pros and cons. • Normalize worries: “Lots of people feel nervous going somewhere new.” • Share success stories. • Ask permission before offering suggestions.	• Marketing such as infographics, leaflets. • Signposting and advice. • A tutorial on what the *Via Connect* platform entails. • Questionnaire to assess interests and needs.
**Preparing to engage** (Considering trying)	• They are open about action but need support. • They want specifics about options. • May need help planning logistics or building confidence	Turn willingness into a practical and achievable first step.	• Co-create an action plan. • Assertively link through introductions and first-visit support with a *Community Connector*. • Identify small first steps, such as visiting a website or having a phone call. • Reduce barriers with transport information, accessibility details and clarity about cost.	• Questionnaire to assess interests and needs. • Use *Via Connect* with a Key Worker or Community Connector.
**Active engagement** (Will attend or have attended)	• They have attended something or will attend something.	Support continuation and early wins.	• Give positive reinforcement. • Troubleshoot early challenges. • Help build routine and social connections. • Compare expectations with real experience.	• Discussions with the Community Connector. • Guided conversation tool, to identify if interests or needs have changed.
**Maintaining engagement** (Attending regularly, staying connected)	• Regular attendance, and a feeling of connectedness • Engagement becomes habitual. • Benefits are clearer, such as confidence, social connection and wellbeing.	Sustain involvement and embed community participation.	• May be considering becoming a Connector themselves.	• Discussions with the Community Connector. • Guided conversation tool, to identify if interests or needs have changed.
**Lapse in engagement** (Stopped engaging)	• Temporary cessation in attendance and/or engagement. • Engagement drops off for many possible reasons, including health, anxiety, life events and mismatched expectations.	Re-engage in a supportive, non-pressured way.	• Normalize relapse as part of change, and not as failure. • Gently explore what happened. • Revisit motivation, goals, and current needs. • Consider alternative community options, if the original did not fit.	• Revisit the guided conversation tool to identify changed interests and needs in this time.

#### 3.3.2 Community connector role description and person specification

Participants identified Community Connectors as key facilitators of engagement with community assets. The role was described as supporting service users to identify, access, and participate in community opportunities, while acting as a bridge between treatment services and the wider community. Participants highlighted the importance of promoting confidence, recovery, and social connection through supportive and informal interactions.

In response to this feedback, the person specification (Table 6) was developed and is intended to be adapted by services as appropriate.

**Table 6:** community connector role and person specification.

Personal attributes
•Friendly, approachable, and supportive manner.
•Ability to build trust and rapport with a wide range of individuals.
•Empathetic, emotionally aware, and non-judgemental.
•Patient and reassuring, particularly when working with individuals who may be anxious or reluctant to engage.
•Open, relatable, and able to communicate authentically.
•Commitment to promoting recovery, inclusion, and community participation.
•Willingness and ability to share appropriate lived experience to inspire and support others.
• Passion for community engagement and asset-based approaches.
Knowledge
•Understanding of the barriers that can affect community participation and social inclusion.
•Awareness of safeguarding, confidentiality, professional boundaries, and appropriate conduct.
•Understanding of equality, diversity, and inclusion principles.
•Knowledge of local community assets, services, and support networks.
•Understanding of recovery-oriented approaches.
• Awareness of models such as the Stages of Engagement and Maslow's Hierarchy of Needs.
Skills and abilities
•Excellent interpersonal and communication skills.
•Ability to engage individuals in informal, supportive conversations.
•Ability to motivate and encourage people to take positive, achievable steps toward community participation.
•Ability to recognize and respond sensitively to resistance, anxiety, or uncertainty.
•Ability to create safe and welcoming environments where individuals feel respected and heard.
•Ability to work effectively with people from diverse backgrounds in a culturally sensitive and inclusive manner.
•Ability to identify individual strengths, interests, and aspirations and use these to support engagement.
•Confidence in establishing and maintaining relationships with community groups and organizations.
•Basic digital skills and ability to use online tools and information systems.
•Ability to facilitate group discussions or drop-in sessions.
•Experience of providing peer support or mentoring.
• Ability to identify and develop future Community Connectors.
Experience
•Experience of working with, supporting, or engaging individuals within community, health, social care, or voluntary sector settings.
•Lived experience of recovery and/or community reintegration.
•Experience of peer mentoring, volunteering, or community development.
• Experience of using digital platforms to support information sharing and engagement.
Role-Specific Responsibilities
•Promote and support engagement with community assets and opportunities.
•Maintain up-to-date information within the Via Connect platform.
•Support service users to identify their interests, needs, and goals through one-to-one and drop-in conversations.
•Accompany service users to community activities where appropriate.
•Develop and maintain positive relationships with local community organizations.
•Gather and share feedback to support the continuous improvement of Via Connect and local asset information.
•Act as a visible ambassador for recovery, community engagement, and social inclusion.
•Participate in all required training and development activities.

#### 3.3.3 Guided conversation tool

A guided conversation tool (Table 7) was proposed as a means of identifying suitable assets and exploring factors that may influence engagement. Discussions emphasized that individuals may not always be able to clearly articulate their needs, and that the benefits of community assets often extend beyond the activity itself.

**Table 7:** Guided conversation tool.

1. What does the service user enjoy doing in their spare time? (*Interests*)
*Allows a gentle start to the conversation and identifies assets that they are accessing currently*.
**2. What matters most to the service user in their daily life and in their recovery journey? (** ***Motivators*** **)**
*Moves from interests toward personal meaning and recovery-related values*.
**3. What matters, and is important, to the service user??**
*Ascertains priorities and core needs and broadens discussion to wider values*.
4. **What aspects of community involvement or support are most important to the service user right now? (*****Needs*****)**
*Categorizes an individual's needs to enable tailored advice*. **a. Psychological: Food, shelter, sleep, clothing. b. Safety: Personal safety, health, financial security, stable housing. c. Love and belonging: Sense of connection, friendship, family connection. d. Self-esteem: Confidence, respect, recognition and giving something back. e. Self-actualisation: Need for personal or skill development, creativity and growth**.
**5. What are the service users' immediate and long-term priorities? (** ***Priorities*** **)**
*Establishes direction and goal setting for engagement*.
**6. What are the service users' fears or concerns about engaging with community assets? (** ***Fears*** **)**
*Identifies barriers and hesitations regarding access*.
**7. Where is the person currently in the Stages of Engagement? (** ***Next steps*** **)**
*Helps to identify what stage a service user is at accessing assets and can direct toward certain assets*.
**Stage of engagement**	**Dates**	**Insert action plan below**
Not ready to engage		
Contemplating engagement		
Preparing to engage		
Active engagement		
Maintaining engagement		
Lapse in engagement		

In response to participant comments, the guided conversation tool aligns with the ABCE framework and draws on Maslow's Hierarchy of Needs (9) to support a holistic understanding of individual needs and aspirations. It also includes space for the guided conversation tool to be completed alongside the stages of engagement framework.

### 3.4 Future development, marketing and visibility

#### 3.4.1 Digital platform development

The digital platform should be introduced early within the service user journey and revisited throughout engagement with services. Initial demonstrations, alongside opportunities for ongoing support, can help increase awareness, confidence and uptake.

The platform should function as a central hub for information on community assets, recovery support, and wider opportunities for participation. To maximize usability, key features should include personalized recommendations, the ability to save resources, search and filtering functions and access to information outside standard service hours. Information should be regularly reviewed to ensure accuracy, relevance and quality.

Accessibility should be a core design principle. Consideration should be given to multilingual resources, captioned video content, clear navigation and user-friendly design. It is also important to recognize that some individuals may experience barriers related to digital literacy, learning needs or personal preferences. As such, digital tools should complement, rather than replace, face-to-face support.

#### 3.4.2 Promotion and visibility

A range of promotional approaches should be used to increase awareness and engagement with the platform. These may include instructional videos, visual displays within service settings and printed materials such as leaflets and posters containing QR codes to facilitate access.

Visible peer support or community engagement roles can play an important part in promoting the platform, supporting individuals to navigate resources and encouraging engagement with community assets. Regular promotion throughout the recovery journey can help ensure that individuals are aware of the support available and are able to access it when it becomes relevant to their needs.

## 4 Discussion

The toolkit presented here is intended as a first attempt at creating a suite of resources to facilitate assertive linkage to community assets for people in recovery. It has been designed to facilitate engagement with Via's digital community asset platform “Via Connect” but is equally applicable in organizations that don't currently have such a platform available to them.

Whilst the toolkit is likely to require further additions and refinements which will be identified through piloting, evaluation exercises and further stakeholder engagement activities, in its current form it provides practitioners and treatment providers with practical tools that can assist in their efforts to engage people in community life.

As stated previously, our Stages of Engagement framework draws on similar processes of behavior change to those identified in the Transtheoretical (TTM) model (34), however our focus is on readiness to engage with community assets rather than on engagement with treatment. The value of applying this approach to our framework lies in the original model's premise that there are multiple complexities inherent in making significant lifestyle changes, and that these can be influenced by employing specific strategies and interventions at the right time in a person's recovery journey (35).

Our adapted framework and accompanying guide therefore provide a useful tool for understanding how and when community-based opportunities can be introduced and explored with service users. The detailed explanations of each stage provided in Table 5 are designed to be used as a training tool and continuous reference guide for staff but should also be used in conjunction with the guided conversation tool presented in part four of the toolkit.

It is important to note that the Stages of Engagement framework does not currently differentiate between the *types* of assets available and whether the service user needs practical support (for example, employment or housing advice) or social engagement (such as access to exercise or art classes); this will be further developed as the framework is piloted within services. In the meantime, the guided conversation tool provides an opportunity to hold a discussion with the service user through the lens of Maslow's hierarchy of needs (9), and to assess where the individual is on that hierarchical journey (10). As with the Stages of Engagement framework, this is not intended to be a one-off task, but a conversation that is revisited regularly as the individual progresses and their wants and needs change.

Guided conversations and brief assessments such as those featured in the toolkit are commonly used in strengths-based approaches as they help the individual identify existing sources of social and recovery capital. Other examples include the ABCE workbook presented by Collinson and Best (6) which encourages individuals to rate their ability to engage with community assets according to four domains: (1) accessibility (2) affordability (3) connectedness to the group and (4) network (whether the network associated with the asset is supportive of recovery or not). This and other tools such as the Recovery Strengths Questionnaire (36) the Recovery Capital Questionnaire (37) and the Social Recovery Capital Questionnaire – Important People and Activities Instrument (38) can all be used in combination with our toolkit to gain a fuller understanding of an individual's needs, if required.

At the heart of our toolkit is the Community Connector person specification which clearly identifies the attributes that anyone facilitating assertive linkage should possess. Whilst this role is similar to social prescribing link workers who support people to access local assets “by identifying personal needs and goals” [(39), p.4], it is imperative that in this context the Community Connector is someone with lived experience and a deep understanding of substance use issues and recovery pathways (40). They also need to be highly visible and easily identifiable to service users by having a regular presence and clothing or badges that make them easy to find and approach for support.

Others have strongly advocated for the importance of a Community Connector role during recovery and the need for connectors to be knowledgeable, well versed in local assets and actively engaged in linking service users to local activities (41). In many services, peer support workers already fulfill this role, acting as a bridge between people in recovery and community resources, activities and social networks (45). However, we have been unable to find any formalized attempt to create a Community Connector person specification for drug treatment and recovery services, and we hope that the inclusion of the description in the toolkit provides a useful basis for future recruitment.

It is also important to consider terminology here; elsewhere Community Connectors have been defined as people who undergo training sessions or workshops that give them the skills to share brief signposting messages with others on an opportunistic basis, often acting in a voluntary capacity (42). However, based on the findings of this study and our underpinning philosophy of assertive linkage, the vision for the role within drug and alcohol treatment services goes far beyond simple signposting duties. As such, Via are currently in the process of developing a robust training package to equip and upskill those taking on the Community Connector role.

Lastly, whilst not all services will have access to a searchable digital platform of community assets, information about local activities is often only available online. Given that our study has highlighted concerns around digital literacy, accessibility and language needs, to avoid worsening inequalities services need to offer support to service users who do not currently have the digital skills to search for community assets on their own (43). In addition, a range of different communication tools should be used to advertise the Community Connector role and the support on offer, ideally through the development of animations, leaflets, promotion at existing events and informal conversations.

In summary, we strongly assert that our toolkit should not be viewed as a standalone guide and that other resources exist that aim to encourage conversations regarding recovery capital through formalized processes. We therefore encourage practitioners to “build their own” approach based on client need and local working practices.

## 5 Conclusion

Sustained recovery from problematic substance use requires more than access to treatment alone. It depends on opportunities to build social connections, develop positive identities, access meaningful activities and increase recovery capital within supportive communities.

Our findings highlight that engagement with community assets is influenced by a range of structural, social and psychological factors, and that successful participation often requires personalized support, trusted relationships and ongoing encouragement. By recognizing readiness for engagement and tailoring support accordingly, services may be better positioned to help individuals overcome barriers, strengthen recovery capital and develop meaningful, lasting community connections.

Whilst the toolkit now requires piloting and formal evaluation to assess implementation, acceptability and outcomes in practice, it offers a promising framework for embedding recovery-oriented, strengths-based approaches within substance use services. More broadly, the principles underpinning the toolkit may have relevance beyond addiction treatment, providing a transferable model for supporting community participation and wellbeing among other populations experiencing social exclusion, stigma or complex life challenges.

## Data Availability

The raw data supporting the conclusions of this article will be made available by the authors, without undue reservation.
